# Influence of Different Damage Patterns of the Stem Taper on Fixation and Fracture Strength of Ceramic Ball Heads for Total Hip Replacement

**DOI:** 10.1155/2020/7542062

**Published:** 2020-05-13

**Authors:** Danny Vogel, Jessica Hembus, Mario Jackszis, Vera Bolte, Rainer Bader

**Affiliations:** Biomechanics and Implant Technology Research Laboratory, Department of Orthopaedics, University Medicine Rostock, Doberaner Straße 142, 18057 Rostock, Germany

## Abstract

**Background:**

Modularity finds frequent application in total hip replacement, allowing a preferable individual configuration and a simplified revision by retaining the femoral stem and replacing the prosthetic head. However, micromotions within the interface between the head and the stem taper can arise, resulting in the release of wear debris and corrosion products. The aim of our experimental study was to evaluate the influence of different taper damages on the fixation and fracture stability of ceramic femoral heads, after static and dynamic implant loading.

**Methods:**

Ceramic ball heads (36 mm diameter) and 12/14 stem tapers made of titanium with various mild damage patterns (intact, scratched, and truncated) were tested. The heads were assembled on the taper with a quasistatic load of 2 kN and separated into a static and a dynamic group afterwards. The dynamic group (*n* = 18) was loaded over 1.5 million gait cycles in a hip wear simulator (ISO 14242-1). In contrast, the static group (*n* = 18) was not mechanically loaded after assembly. To determine the taper stability, all heads of the dynamic and static groups were either pulled off (ASTM 2009) or turned off (ISO 7206-16). A head fracture test (ISO 7206-10) was also performed. Subsequent to the fixation stability tests, the taper surface was visually evaluated in terms of any signs of wear or corrosion after the dynamic loading.

**Results:**

In 10 of the 18 cases, discoloration of the taper was determined after the dynamic loading and subsequent cleaning, indicating the first signs of corrosion. Pull-off forces as well as turn-off moments were increased between 23% and 54% after the dynamic loading compared to the unloaded tapers. No significant influence of taper damage was determined in terms of taper fixation strength. However, the taper damage led to a decrease in fracture strength by approximately 20% (scratched) and 40% (truncated), respectively.

**Conclusion:**

The results suggest that careful handling and accurate manufacturing of the stem taper are crucial for the ceramic head fracture strength, even though a mild damage showed no significant influence on taper stability. Moreover, our data indicate that a further seating of the prosthetic head may occur during daily activities, when the resulting hip force increases the assembly load.

## 1. Introduction

Today, a modular taper-head connection is considered the state of the art in total hip replacement. In general, modular taper connections combine an easy assembly of the ball head, while enabling the removal of the head and cup insert in case of a revision surgery, offering bone preservation. Further, the modularity provides individual configuration due to different implant geometries and materials and their combination. Besides these benefits, some disadvantages can arise.

In total hip replacement (THR), micromotions between the ball head and the stem taper may occur, when the fixation strength is not strong enough to withstand the resulting torsional and bending moments during daily activities. Thereby, the interface (trunnion) between the prosthetic head and taper is a potential source for wear debris and corrosion [1–10].

The combination of wear particles and corrosion can be related to a multitude of biological reactions, including adverse local tissue reactions like inflammation, pseudotumors, osteolysis, aseptic implant loosening, and aseptic lymphocytic vasculitis-associated lesion (ALVAL) [3, 4, 11–15]. Moreover, abrasive wear particles can migrate into the joint gap and potentially lead to highly increased wear of the articulating implant components, called third-body wear [16].

The influence of various factors like assembly load, material combinations, and contamination on the taper stability was investigated in several studies [4, 17–21]. However, the effect of different stem taper damages on the taper stability has not been investigated so far.

Further, an inhomogeneity at the taper surface due to wear or damage results in irregular stress distribution and stress peaks, being potentially critical in particular for ceramic heads in terms of brittle fracture [22–24]. In a standard case of revision surgery, an adapter sleeve inside the ceramic head is therefore used, when the femoral stem is not exchanged, to avoid high stress peaks. However, damage of the stem taper like scratches from surgical instruments occurring during primary total hip replacement cannot be ruled out, and therefore, ceramic heads might be combined with damaged tapers without adapter sleeves as well.

Therefore, the aim of our experimental study was to evaluate the influence of different taper damages on the fixation and fracture stability of ceramic femoral heads. Therefore, the effect of two defined damage patterns on the taper connection and fracture strength of ceramic heads was investigated after static and dynamic implant loading.

## 2. Materials and Methods

Thirty-six stem tapers (Ti-6Al-4V, 12/14 taper, ridge height 22 *μ*m, ridge spacing 130 *μ*m, taper length 18.5 mm) were used in the present study. While twelve tapers remained as manufactured (intact: I), two different damage patterns were applied to the remaining tapers, resulting in three different groups of tapers.

**Figure 1:**
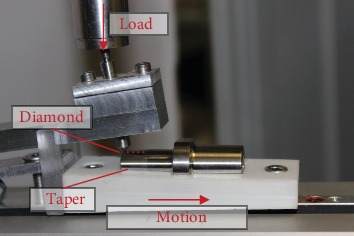
Setup to apply a scratch to the taper surface.

The first damage pattern was a defined scratch at the taper surface perpendicular to the ridge structure, which could possibly occur during the implantation procedure (e.g., from careless instrument handling). The scratches were produced using a self-constructed scratch test setup with a diamond point loaded with 400 N by a universal testing machine Zwick Z050 (ZwickRoell GmbH & Co. KG, Ulm, Germany) (Figure 1). The scratches had a mean depth of 73 *μ*m and a width of 411 *μ*m.

**Figure 2:**
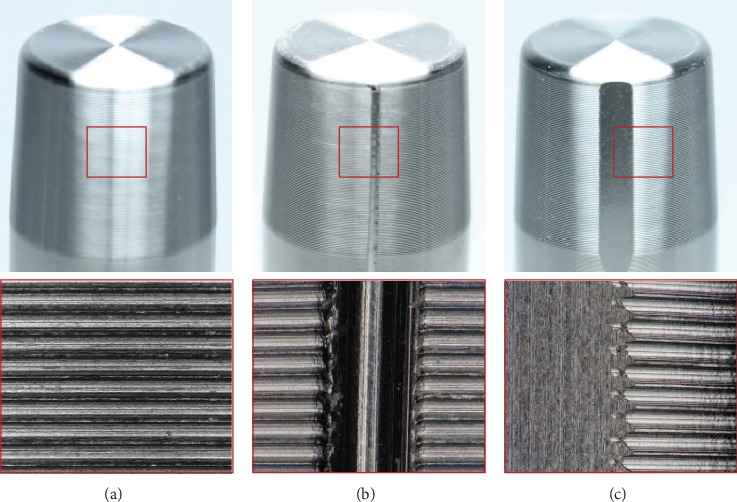
Depiction of the damage patterns of the stem tapers: (a) intact, (b) scratched, and (c) truncated.

The second damage pattern was chosen to represent a truncated taper that could occur due to abrasion over time [6]. This damage pattern was realized by a 0.1 mm deep groove in the axial direction of the stem taper, resulting in an approximate volumetric material loss 1.8 mm^3^. The different taper damage patterns are shown in Figure 2.

**Figure 3:**
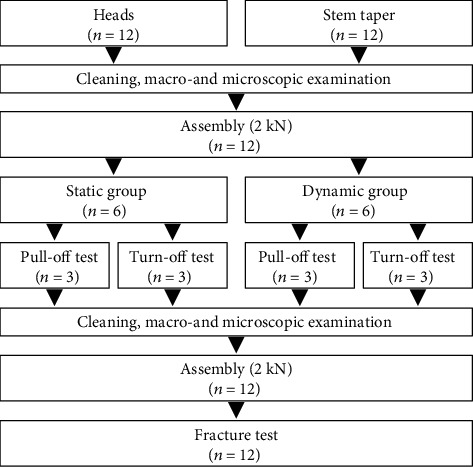
Scheme of the experimental test design. This procedure was carried out for each of the three groups of tapers resulting in a total of 36 heads and tapers.

A total of thirty-six alumina-toughened zirconia heads (36 mm diameter, 12/14 taper, Mathys Orthopaedie GmbH, Mörsdorf, Germany) were assembled to the tapers with a quasistatic load of 2 kN (displacement rate of 0.05 mm/s) in accordance with ASTM 2009 [25] using the universal testing machine Zwick Z050 (ZwickRoell GmbH & Co. KG, Ulm, Germany). Before the assembly process, the components were cleaned according to ISO 14242-2 [26] to remove all contaminants. Thereafter, the assembled heads were divided into two subgroups. In the case of the first group, the pull-off (PO) and turn-off (TO) tests were carried out after the assembly without further applied loads (static group: ST). The second subgroup was dynamically loaded over 1.5 million cycles in a standard hip simulator (EndoLab GmbH, Rosenheim, Germany) according to ISO 14242-1 [27], with load of the normal gait cycle between 0.3 and 3.0 kN and a frequency of 1 Hz (dynamic group: DYN). Due to the dynamic loading in the hip simulator, the taper connection was subjected to frictional and additional bending moments.  Figure 3 shows the test groups and the experimental design.

### 2.1. Macroscopic Examination

Before and after the dynamic loading, the taper surfaces were macroscopically examined. For the macroscopic examination, all specimens were photographed. The taper surfaces were evaluated according to severity as per Goldberg et al. [10], and the damage profiles (ring-shaped, opposite-sided, and one-sided diffused) were classified according to Cook et al. [6] after the dynamic loading.

### 2.2. Pull-Off Test

**Figure 4:**
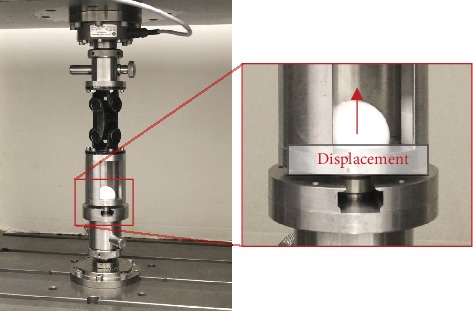
Setup for the pull-off test using a universal testing machine.

The pull-off test of the femoral heads was carried out through the universal testing machine Zwick Z050 (ZwickRoell GmbH & Co. KG, Ulm, Germany). The taper was clamped in a fixation device, while the head was pulled off the taper with a constant displacement rate of 0.05 mm/s (Figure 4). The test was terminated when a sudden drop of the load was detected, with the peak load giving the pull-off force. The pull-off forces were measured in accordance with ASTM 2009 [25].

### 2.3. Turn-Off Test

**Figure 5:**
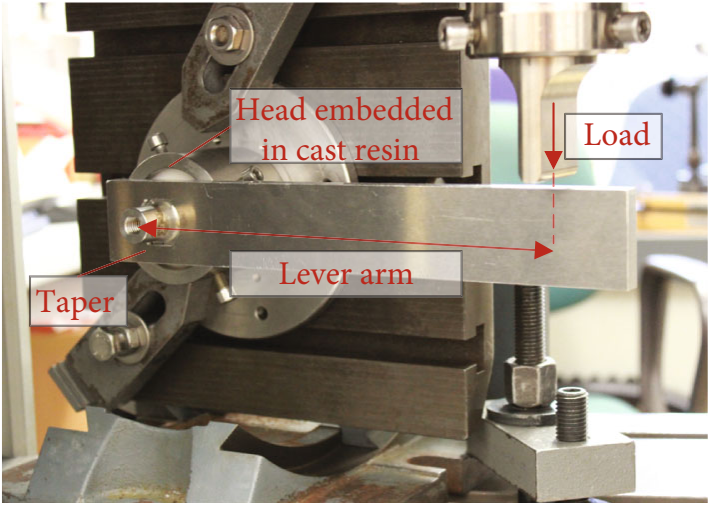
Setup for the turn-off test using a universal testing machine.

The turn-off tests of the femoral heads were carried out through the universal testing machine Zwick Z050 (ZwickRoell GmbH & Co. KG, Ulm, Germany) as well. Turn-off moments were measured based on ISO 7206-13 [28]. The ceramic heads were therefore embedded in two-component cast resin (Component 1: RenCast FC 52 BD Polyol; Component 2: RenCast FC 52/53 BD Isocyanate; Huntsman AG, Salt Lake City, UT, USA), and the taper was orientated horizontally. A lever arm with a length of 200 mm was fixed to the taper, horizontally aligned, and loaded perpendicularly with a load rate of 1 N/s (Figure 5). Thus, a torsional moment was applied to the taper, loosening the taper-head connection. The test was terminated when a sudden drop of the load occurred or an angle limit of 20° was reached. Maximal torsional moments were calculated from the recorded load and the lever arm length.

### 2.4. Fracture Test

The fracture loads of the ceramic heads were determined in accordance with ISO 7206-10 [29]. The heads were loaded through an indenter with a copper ring placed between the head and the indenter using a universal testing machine (Instron 8502, Instron, Norwood, MA, USA). The load was increased with a displacement rate of 0.04 mm/s until a ceramic fracture occurred or a plastic deformation of the taper was detected.

### 2.5. Statistics

For statistical evaluation, IBM® SPSS® (Statistics Version 25, IBM Corporation, Armonk, NY, USA) was used to run two different tests. The Mann-Whitney *U* test was used to evaluate differences in fixation stability (PO, TO) and fracture load between the unloaded (ST) and dynamically loaded (DYN) implant specimens. To analyse differences resulting from the altered taper geometry, the Kruskal-Wallis test was applied. When a significant difference was obtained via the Kruskal-Wallis test, the Mann-Whitney *U* test was applied to detect where the differences were situated, by making pair-wise comparisons. Data was presented as the mean value ± standard deviation, and *p* values and effect size were determined. *p* values < 0.05 were considered significant, and effect sizes of 0.1 ≤ *r*_pb_ < 0.3 were considered small, 0.3 ≤ *r*_pb_ < 0.5 medium, and *r*_pb_ ≥ 0.5 large.

## 3. Results

### 3.1. Macroscopic Examination

**Figure 6:**
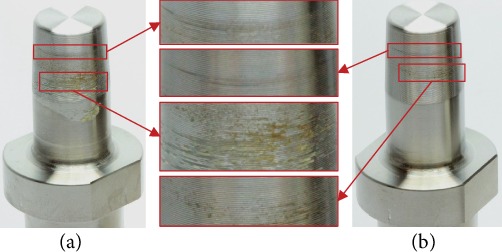
Discoloration of an intact taper after dynamic loading (left) and after cleaning (right).

After removal of the implant specimens from the hip wear simulator, all dynamically loaded stem tapers were covered in a mixture of creamy-colored dried serum and had darker orange to brown discolored areas. The serum and most parts of the darker discolorations were removed after the cleaning protocol. However, in 10 of 18 stem tapers (4x intact taper, 4x scratched, and 2x truncated), discolorations were observed after cleaning (Figure 6). No deformations, additional scratches, or abrasions were macroscopically visible, and therefore, the damage pattern conformed to Goldberg score 1.

### 3.2. Pull-Off Test

**Figure 7:**
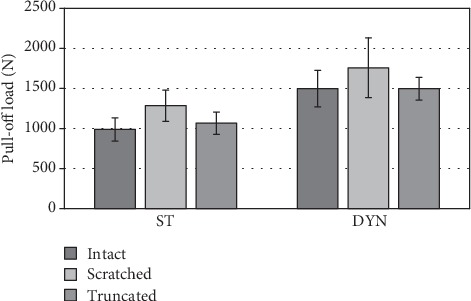
Differences of the pull-off forces in dependency on taper damage and the loading condition. ST: statically loaded; DYN: dynamically loaded.

The pull-off forces increased between 36.8% and 51.6% (effect size 0.45-0.80) after the dynamic loading. In the statically loaded group, the scratched tapers showed the highest pull-off forces (1,284.8 N ± 194.9 N), followed by the truncated (1,065.4 N ± 137.9 N) and intact (987.6 N ± 144.9 N) tapers. In the dynamically loaded groups, the scratched tapers showed the highest pull-off forces (1,757.9 N ± 373.7 N) followed by the intact (1,497.5 N ± 227.0 N) and truncated (1,496.0 N ± 141.5 N) tapers. None of the differences were statistically significant (Figure 7).

### 3.3. Turn-Off Test

**Figure 8:**
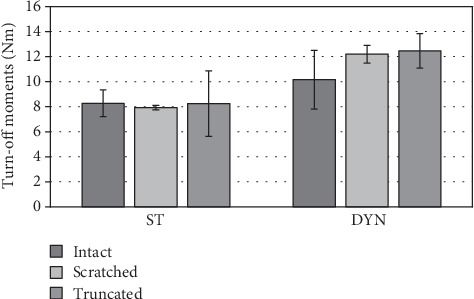
Differences of the turn-off moments in dependency on taper damage. ST: statically loaded; DYN: dynamically loaded.

Apart from the pull-off forces, the turn-off moments increased between 22.8% and 53.8% (effect size 0.45-0.80) after the dynamic loading. In the statically loaded group, the intact tapers showed the highest turn-off moments (8.3 Nm ± 1.1 Nm), followed by the truncated (8.2 Nm ± 2.6 Nm) and scratched (7.9 Nm ± 0.2 Nm) tapers, respectively. In the dynamically loaded group on the other hand, the truncated tapers (12.5 Nm ± 1.4 Nm) showed the highest turn-off moments followed by the scratched (12.2 Nm ± 0.7 Nm) and intact (12.2 Nm ± 2.3 Nm) tapers. None of the differences were statistically significant (Figure 8).

### 3.4. Fracture Loads

Higher fracture loads were determined in the case of the intact tapers (static: 162.8 kN; dynamic: 177.6 kN), compared to the damaged tapers. The fracture load was decreased by about 15.0% to 20.9% in the case of the scratched tapers and by about 38.9% to 43.1% in the case of the truncated tapers. The differences were statistically significant and had a large effect size, when the truncated tapers were compared to the intact or scratched tapers. When the scratched tapers were compared to the intact tapers, a medium effect was determined, but statistical significance was not observed.

**Figure 9:**
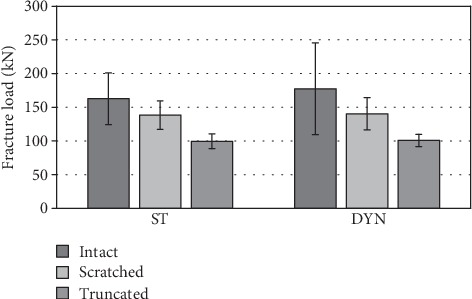
Differences of the fracture loads in dependency on taper damage and the loading condition. ST: statically loaded; DYN: dynamically loaded.

Comparing statically and dynamically loaded stem tapers, the dynamically loaded tapers showed a 1.4% to 9.1% increased fracture strength. This finding was not statistically significant (Figure 9).

**Figure 10:**
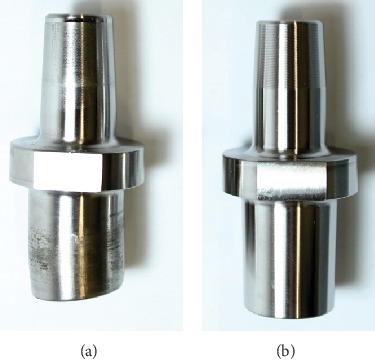
Comparison of a plastic deformed (a) and intact (b) stem taper.

However, in some fracture tests, a plastic deformation of the stem taper was detected before the ceramic femoral head fractured (Figure 10). A plastic deformation was determined in the case of 11 stem tapers (6x intact, 4x scratched, and 1x truncated). These measurements were excluded from the result analysis.

## 4. Discussion

Stem taper damage and wear are complex problems in modular THR, caused by multiple causal factors. Damage of the stem taper may lead to adverse effects on the fracture strength of ceramic heads and should be avoided, to prevent possible early implant failure [22, 23]. Moreover, it was shown in previous studies that contaminations of the taper-head interface result in a lowered fixation and fracture strength [22, 30]. However, the effect of taper damages on the fixation strength has not been investigated in experimental studies so far.

Hence, in the present study, we investigated the effects of stem taper damage patterns on the taper-head fixation and fracture strength of ceramic ball heads. The generated damage patterns simulated either a scratch that may be produced by a surgical instrument during THR surgery or a truncated taper representing a possible one-sided wear pattern occurring over time.

Our study is constrained by certain limitations. First of all, the experimental study is limited by its small sample size of three for each group leading to a small statistical significance and effect size, harboring the risk of over- or underestimation of the results. Therefore, the results were critically assessed with respect to previous findings.

Moreover, even so, the chosen displacement rate of 0.05 mm/s to pull off the heads is in accordance with ASTM F2009; a displacement-controlled procedure might lead to an underestimation of the pull-off forces due to the limited data acquisition rate of the testing machine and the loosening of the head occurring in a matter of less than a millisecond. However, our determined results and ratios between assembly loads and pull-off loads were in tune with the literature and therefore are considered to be plausible [4, 31].

Further, only a short period of 1.5 million cycles of dynamic loading was applied in our study, which corresponds to about 1.5 to 2 years of implantation time, representing only a short period in the average lifespan of a THR [32, 33]. However, it cannot be ruled out that an influence of taper damage will occur after a longer test period ex vivo, as corrosion initialization takes time *in vivo* [34].

Further, taper stability and wear are affected by lubrication, if the lubricant enters the crevice between the implant components. Since further investigations in our laboratory revealed significant differences of rheological properties of bovine serum, Ringer's solution, and patient synovia, an effect of the chosen lubrication on taper stability and wear cannot be excluded.

The amount of stem taper abrasion could not be determined in our study. However, a grey discoloration was detected inside the head taper, indicating that abrasive wear particles were transferred from the stem taper to the ceramic head. Therefore, the amount of wear at the stem taper should be assessed in further investigations.

Despite these limitations, the strength of this study is the large variety of tests carried out to evaluate the relation between superficial damage patterns, visual signs of wear and corrosion, taper stability, and fracture strength of ceramic ball heads.

Stem taper damage was analysed by visual examination in our current study. Serum residues were observed on the stem taper surface after disassembly, indicating that fluid had entered the crevice. Slight dark and dull ring-shaped discolorations were detected on nearly every dynamically loaded taper, which were assumed to be a mild form of corrosion. According to Kurtz et al. [9], the determined amount of damage can be classified as grade 1, a minimal fretting or corrosion on less than 10% of the taper surface, and according to Goldberg, as grade 1 (“none”) or grade 2 (“mild”) [10]. To check whether the discoloration represents corrosion, alternative ways of surface analysis, like energy dispersive X-ray spectroscopy (EDX), should be considered in further investigations.

In the present study, each specimen running 1.5 million cycles was exposed to constant dynamic loading over three weeks in tempered bovine serum. This can be compared to approximately 1.5 to 2 years *in vivo* and is therefore a short period, compared to the time that THRs last *in vivo* [32, 33]. Collier et al. found at 90% of explanted THR components made of different alloys (Ti-stem/CoCr-head) a 10% or less corroded surface in an average implantation time of 29.1 months. All implants with a corroded taper surface greater than 10% had been implanted for in average 46.3 months [34]. Taking this into consideration, five million cycles as suggested in ISO 14242-1 might have led to stronger signs of corrosion and a better comparability to clinical studies investigating corrosion. Moreover, it is known that the use of ceramic femoral heads on stem tapers made of titanium reduces corrosion and taper damage, compared to metallic heads [9, 35, 36]. Therefore, it is possible that taper damage leads to increased corrosion and wear, when the same tests are performed using metallic heads. Another important factor regarding taper corrosion is the bending moment arising during loading. One offset length of femoral heads was only considered in our present study; however, larger offset heads are associated with increased bending moments and therefore increased taper corrosion [36].

To evaluate the influence of different damage patterns on the taper fixation strength, the pull-off force and turn-off moments were determined. Several studies show that the taper stability is, regardless of material and component geometry, highly related to the assembly load. While Danoff et al. found pull-off forces averaging 45% of the assembly load, Rehmer et al. showed an average pull-off force of 55% [4, 31]. This is in tune with the current results finding pull-off forces of 54% (intact: 49%, scratched: 64%, and truncated: 53%) of assembly load in the case of the not dynamically loaded specimen. After the dynamic loading, the relation of the pull-off force to the assembly load was increased, with the mean pull-off force being 79.2% of the assembly load of 2 kN (intact: 74.9%, scratched: 87.9%, and truncated: 74.8%). This is explained by the maximal load of 3 kN during the dynamic loading acting as an additional and repetitive assembly load, resulting in a further seating of the head and increasing the pull-off force. When the relation of the pull-off force to the dynamic load of 3 kN was compared, it was nearly the same as in the static group, with a mean relation of 53% (intact: 50%, scratched: 59%, and truncated: 50%). This is in line with previous studies, which showed a linear relationship between assembly and pull-off force [4, 18, 31, 37]. In further investigations, an assembly load above the maximum load during dynamic testing has to be analysed in order to determine whether the dynamic load has an influence on the fixation strength of the taper connection.

Additionally, the turn-off moments were evaluated as they are more representative of the risk of *in vivo* failure, due to frictional torques.

Similar to the pull-off force, the turn-off moments were 23% (intact), 53% (truncated), and 54% (scratch) higher after the dynamic loading, compared to the unloaded reference. These results underline the assumption that the dynamic loading with a maximum load above the assembly load initiates an additional seating of the head, resulting in higher fixation strength.

The determined turn-off moments between 7.9 Nm and 8.2 Nm in the unloaded and 10.2 Nm and 12.5 Nm in the dynamically loaded group were in good agreement compared to the results found by Rehmer et al. [4]. Deviations can result from various reasons like the chosen taper geometry or taper surface; moreover, we used a static assembly load compared to the dynamic impact chosen by Rehmer et al.

When the arising moments resulting from the frictional torques during daily activities exceed the torsional strength of the taper connection, micromotions might occur, subsequently resulting in fretting, corrosion, and wear at the taper-head interface [2, 36, 38]. Subsequently, abrasion at the taper surface may lead to the release of wear particles with subsequent biological reactions, including adverse local tissue reactions like inflammation, osteolysis, aseptic loosening, and ALVAL [3, 4, 11–15]. Therefore, the determined turn-off moments have to be compared to moments of acting *in vivo*, to evaluate the safety of the taper connection. As the resulting moments around the taper axis have not been measured *in vivo* so far, the moments have to be compared to those measured in experimental test setups. Bishop et al. determined frictional moments in the case of a ceramic-on-ceramic bearing (head diameter 28 mm) between 2.0 Nm and 2.9 Nm, indicating that the static assembly load of 2 kN is strong enough to avoid micromotions due to the frictional moments [39]. However, the same study showed an influence of various factors like head material, head diameter, and lubricant on the frictional moments [39]. In the case of a fully dried-out bearing, the frictional moments are most likely to increase even further. Therefore, it cannot be generally stated that an assembly load of 2 kN is high enough in all means of THR.

Although it was expected that the damage patterns selected in our study would lead to reduced fixation strength, no significant effect of the tested taper damage on the pull-off forces or turn-off moments was found. In contrast, we could show that the fixation strength increases due to the dynamic loading, when the maximal dynamic load exceeds the applied assembly load.

In the present study, a significant influence of the tested taper damages was determined. A damage of the taper led to reduced fracture loads up to 44% compared to undamaged tapers, whereby the fracture load of the truncated tapers was the lowest (100 kN), followed by that of the scratched taper (134 kN). This may be explained by the increased area of damage in the case of the truncated tapers, which is up to 5.5% of the contact surface between the ceramic head and the taper. Therefore, stress is distributed on a smaller surface, leading to an increase of contact forces and subsequently higher principal stresses. In particular, the chamfer edges from which the fracture originated are critical in terms of stress increase.

In previous studies, the influence of different damage patterns and contaminations on the fracture strength of ceramic heads was evaluated [22, 23]. Wuttke et al. [23] examined the effect of emerged edges, which can arise from a careless clash on the taper, by placing titanium wires (*Ø* 0.4 mm and *Ø* 0.25 mm) between the taper and the head. These titanium wires led to a reduction in fracture strength of 46% to 67% [23]. In contrast, the ridges arising from the applied scratches were considerably low in our experiments, explaining the reduced influence on the fracture strength. In conclusion, the height of the simulated ridge is essential to the fracture strength by determining the extent of stress increase, and higher ridges should be considered in further investigations.

Weisse et al. examined the influence of truncated tapers on the fracture strength [22] and determined a reduction of fracture strength by about 26%, when the taper was truncated. This influence found is lower compared to our present study, in which the same truncation (0.1 mm) led to a reduction of 40%. However, the fracture loads were lower compared to our study (intact taper: 87.6 kN-97.5 kN; truncated: 67.9 kN-68.7 kN), which can be explained by the smaller head size (*Ø* 28 mm), different neck length, and the ceramic material (A_2_O_3_) [22].

## 5. Conclusion

In our present study, the ceramic heads could be well fixed on damaged tapers and the fracture loads were two to three times higher than the required fracture load of 46 kN and physiological loads acting at the hip *in vivo* in worst case scenarios like stumbling. However, a variety of factors like the head size, ceramic material, neck length, and altered loading conditions, like a physiological loading angle (40°), influence the fracture strength and can lead to reduced fracture loads below the required 46 kN [22, 23, 40, 41]. Further, we determined a subsequent head seating under dynamic loading, when the load during a gait cycle exceeds the assembly load.

Kim et al. postulated that 36 mm ceramic heads can be used in revision surgery without adapter sleeves [42]. However, despite the adequate head fixation on damaged tapers in our experimental tests, we recommend the use of adapter sleeves, which are known to reduce the ceramic fracture risk [43, 44].

## Data Availability

The experimental data used to support the findings of this study are available from the corresponding author upon request.
